# Supplementary material for: Analytical and clinical validation of a multiplex PCR assay for detection of Neisseria gonorrhoeae and Chlamydia trachomatis including simultaneous LGV serotyping on an automated high-throughput PCR system
Source: Microbiol Spectr. 2024 Feb 12;12(3):e02756-23. doi: 10.1128/spectrum.02756-23 (PMC10913481; doi:10.1128/spectrum.02756-23)
Supplement: Supplemental material — Fig. S1 and Tables S1 to S8. [file spectrum.02756-23-s0001.docx]

**Analytical and clinical validation of a multiplex PCR assay for detection of Neisseria gonorrhoeae and Chlamydia trachomatis including simultaneous LGV serotyping on an automated high-throughput qPCR system.**

Lisa Sophie Pflüger^a^, Dominik Nörz^a^, Moritz Grunwald^a^, Susanne Pfefferle^a^, Katja Giersch^a^, Martin Christner^a^, Beatrice Weber^a^, Martin Aepfelbacher^a^, Holger Rohde^a^, Marc Lütgehetmann^a,b^

**Affiliations**:

^a^ Institute of Medical Microbiology, Virology and Hygiene, University Medical Center Hamburg-Eppendorf (UKE), Hamburg, Germany.

^b^ German Center for Infection Research (DZIF), Hamburg-Lübeck-Borstel-Riems Site, Hamburg, Germany

**Corresponding author**: Marc Lütgehetmann, m.luetgeh@uke.de

**Supplementary material**

1. **Figures**

**
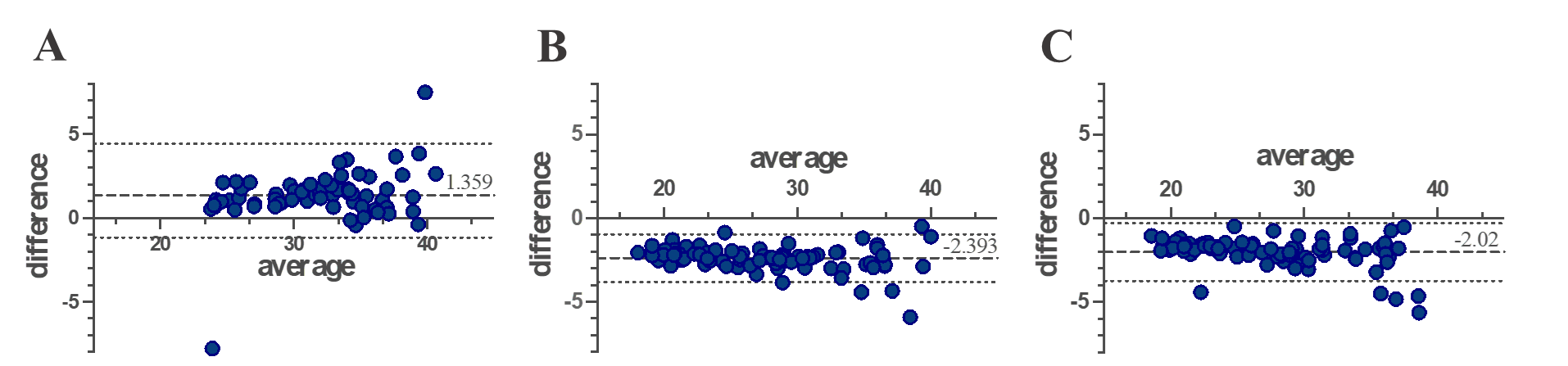
**

**Supplementary figure 1). Bland-Altman plots:** Bland-Altman analysis of test results of the LDT (UC_CTNG) and CE-IVD (cobas CT/NG, Roche) assays detecting **(A)** CT serovars A-L3, **(B)** NG (LDT target: opa) and **(C)** NG (LDT target: porA). The mean of the bias is plotted (dashed gray line) as well as the corresponding 95% confidence intervals (dotted gray lines, +/- 1.96 SD).

Abbreviations: CE-IVD, CE-trademarked in vitro diagnostics; ct, cycle threshold, CT, Chlamydia trachomatis; LDT, laboratory developed test; NG, Neisseria gonorrhoeae; UC_CTNG; Utility Channel Chlamydia trachomatis and Neisseria gonorrhoeae.

1. **Tables**

**Supplementary table 1.)** **Comparison of technical performance in swabs collected from different body sites:** Clinical samples (swabs, all collected and stored in eSwab medium, Copan) were pooled and tested negative for *CT* and *NG* by qPCR. Next, matrix pools were diluted with Cobas PCR media (Roche; ratio: 50:50). Using highly clinical samples which were quantified using digital PCR, dilution series were tested with the new UC_CTNG (6 steps, 8 repeats per step) for each matrix pool seperatly.

* target for dPCR quantification: pmpH, LGV-specific probe (single-copy gene)

** target for dPCR quantification: opa (multi-copy gene)

Abbreviations: CT, *Chlamydia trachomatis*; conc., concentration; dcp, copies as quantified by digital PCR; NG, *Neisseria gonorrhoae*; PCR, polymerase chain reaction; UC_CTNG, Utility channel test for detection of *Chlamydia trachomatis* and *Neisseria gonorrhoae.*

**Supplementary table 2). Inter- and intrarun variability:** Precision was examined over the course of three days. On the first day each sample (eSwab) was tested in technical triplicates, followed by testing of one replicate of each sample on the second and third day. All samples were tested on the same device.

Abbreviations: CI, confidence interval; CT, Chlamydia trachomatis; ct, cycle threshold; n/a, not applicable; NG, Neisseria gonorrhoeae; SD, standard deviation.

**Supplementary table 3).**

**Exclusivity**: A set of clinical samples and isolates (n=42) used to confirm exclusivity of the newly established multiplex PCR assay and the corresponding test results of the currently used in-house method at the time of sample collection. No false positive test result occurred.

Abbreviations: CFU, colony forming units; cop, copies.

**Supplementary table 4). Discordant test results:** Ct values of discordant test results between the laboratory developed multiplex PCR assay (LDT) and a CE-IVD assay (CT/NG, Roche, Rotkreuz, Switzerland) of a set of clinical samples (n=319).

Abbreviations: CE-IVD, CE-trademarked in vitro diagnostics; CT, Chlamydia trachomatis; LDT, laboratory developed test; NG, Neisseria gonorrhoeae; PCR, polymerase chain reaction; s., serovars.

**Supplementary table 5). Evaluation of clinical performance:** To analyze the clinical performance of the newly established UC_CTNG assay under routine conditions, test results of all clinical specimens within a seven-month time period were evaluated.

* detected by the non-LGV specific probes (nonLGV-probe_01 and nonLGV-probe_02, see table 1) that bind to the pmpH and cryptic plasmid gene, respectively. Both probes are labeled with the same fluorophore, hence both signals are detected in the same channel.

** detected by the LGV-specific probe that hybridizes to the junction area of a deletion region that is strictly present in LGV strains. LGV strains are detected in a separate channel.

**Supplementary table 6). Discordant test results in oropharyngeal swabs:** N=4,298 clinical specimens that were send in for testing of CT and *NG* at our center within a seven-month time period (01.11.22 – 31.05.23) were retrospectively evaluated. Within the analyzed samples n=1,170 oropharyngeal swabs could be identified with 41/1,170 testing positive for both NG targets and 15/1,170 yielding a discordant result.

Abbreviations: CT, Chlamydia trachomatis; ct, cycle threshold; NG, Neisseria gonorrhoeae.

**Supplementary table 7). PCR setup:** PCR profile as programmed using the Cobas omni Utility Channel Software. The sequence of the RNA target used as a spiked-in internal control as well as the sequences of the primers and probe for specific amplification of the internal control are not disclosed by the manufacturer. The relative fluorescence increase (RFI) can be set using the respective software and is used for automatic calling of qualitative test results.

* detection of the LGV-specific target within the pmpH gene (the probe hybridizes to the junction area of a deletion region that is strictly present in LGV strains).

** detection of the non-LGV specific target within the pmpH gene (the probe hybridizes within the deletion area that is strictly present in LGV strains).

Abbreviations: CT, Chlamydia trachomatis; LGV, lymphogranuloma venereum; n/a, not applicable; NG, Neisseria gonorrhoeae; no., number; RFI, relative fluorescence increase.

**Supplementary table 8). Assembly of the master mix:** Volumes are listed for the assembly of the master mix to load one reagent cassette that allows for 192 reactions. A total of 10 ml is needed to fill the cassette. The UC MMX-R2 (Roche, Rotkreuz, Switzerland) includes sequence specific primers and probe for selective amplification of the internal control that is spiked-in automatically during extraction. Concentrations correspond to the final concentration within the reaction mix (including the eluate).

Abbreviations: conc., concentration; UC MMX-R2, Utility Channel Master Mix Reagent 2; fwd, forward; PCR, polymerase chain reaction; plsmd, plasmid; rev, reverse.
